# Metabolic risk factors for esophageal squamous cell carcinoma and adenocarcinoma: a prospective study of 580 000 subjects within the Me-Can project

**DOI:** 10.1186/1471-2407-14-103

**Published:** 2014-02-18

**Authors:** Björn Lindkvist, Dorthe Johansen, Tanja Stocks, Hans Concin, Tone Bjørge, Martin Almquist, Christel Häggström, Anders Engeland, Göran Hallmans, Gabriele Nagel, Håkan Jonsson, Randi Selmer, Hanno Ulmer, Steinar Tretli, Pär Stattin, Jonas Manjer

**Affiliations:** 1 Institute of Medicine, Sahlgrenska Academy, University of Gothenburg, Gothenburg, Sweden; 2 Department of Surgery, Skåne University Hospital, Malmö and Lund University, Malmö, Sweden; 3 Department of Surgical and Perioperative Sciences, Urology and Andrology, Umeå University, Umeå, Sweden; 4 Agency for Preventive and Social Medicine, Bregenz, Austria; 5 Department of Global Public Health and Primary Care, University of Bergen, Bergen, Norway; 6 Norwegian Institute of Public Health, Oslo/Bergen, Norway; 7 Department of Surgery, Skåne University Hospital, Lund and Lund University, Lund, Sweden; 8 Department of Public Health and Clinical Medicine, Nutritional Research, Umeå University, Umeå, Sweden; 9 Institute of Epidemiology and Medical Biometry, Ulm University, Ulm, Germany; 10 Department of Radiation Sciences, Oncology, Umeå University, Umeå, Sweden; 11 Department of Medical Statistics, Informatics and Health Economics, Innsbruck Medical University, Innsbruck, Austria; 12 Cancer Registry of Norway, Institute of Population-based Cancer Research, Oslo, Norway; 13 The Malmö Diet and Cancer Study, Skåne University Hospital, Malmö, Sweden; 14 Department of Internal Medicine, Division of Gastroenterology and Hepatology, Sahlgrenska University Hospital, SE-413 45 Gothenburg, Sweden

**Keywords:** Esophageal cancer, Esophageal adenocarcinoma, Esophageal squamous cell carcinoma, Obesity, Hypertension

## Abstract

**Background:**

Obesity is associated with an increased risk of esophageal adenocarcinoma (EAC) and a decreased risk of esophageal squamous cell carcinoma (ESCC). However, little is known about the risk of EAC and ESCC related to other metabolic risk factors. We aimed to examine the risk of EAC and ESCC in relation to metabolic risk factors, separately and combined in a prospective cohort study.

**Methods:**

The Metabolic Syndrome and Cancer cohort includes prospective cohorts in Austria, Norway and Sweden, with blood pressure, lipids, glucose and BMI available from 578 700 individuals. Relative risk (RR) for EAC and ESCC was calculated using Cox’s proportional hazards analysis for metabolic risk factors categorized into quintiles and transformed into z-scores. The standardized sum of all z-scores was used as a composite score for the metabolic syndrome (MetS).

**Results:**

In total, 324 histologically verified cases of esophageal cancer were identified (114 EAC, 184 ESCC and 26 with other histology). BMI was associated with an increased risk of EAC (RR 7.34 (95% confidence interval, 2.88-18.7) top versus bottom quintile) and negatively associated with the risk of ESCC (RR 0.38 (0.23-0.62)). The mean value of systolic and diastolic blood pressure (mid blood pressure) was associated with the risk of ESCC (RR 1.77 (1.37-2.29)). The composite MetS score was associated with the risk of EAC (RR 1.56 (1.19-2.05) per one unit increase of z-score) but not ESCC.

**Conclusions:**

In accordance with previous studies, high BMI was associated with an increased risk of EAC and a decreased risk of ESCC. An association between high blood pressure and risk of ESCC was observed but alcohol consumption is a potential confounding factor that we were not able to adjust for in the analysis. The MetS was associated with EAC but not ESCC. However this association was largely driven by the strong association between BMI and EAC. We hypothesize that this association is more likely to be explained by factors directly related to obesity than the metabolic state of the MetS, considering that no other metabolic factor than BMI was associated with EAC.

## Background

Esophageal cancer is the eighth most common cancer and the sixth most common cause of cancer-related mortality worldwide [1]. Esophageal cancers can be divided into esophageal squamous cell carcinoma (ESCC) and esophageal adenocarcinoma (EAC). These two cancer types have distinct epidemiological characteristics [2]. The incidence of EAC has risen dramatically in Western countries during the last decades, particularly among white males [3,4], while the incidence of ESCC has been stable or slightly decreasing [2]. Obesity, gastro-esophageal reflux disease and tobacco smoking have been demonstrated to be risk factors for EAC while Helicobacter pylori seropositivity seems to have a protective effect [5]. Established risk factors for ESCC are tobacco smoking, alcohol consumption, low intake of fruits and vegetables and low socioeconomic status [5].

The metabolic syndrome (MetS) is a cluster of metabolic risk factors, including obesity, hypertension, insulin resistance/hyperglycemia and dyslipidemia that has been shown to be associated with cardiovascular disease [6,7]. There is now accumulating evidence that the MetS also may be an important risk factor for several specific cancers as well as overall cancer mortality [8]. A recent meta-analysis has reported an increased risk for liver, colorectal, bladder, pancreatic, breast and endometrial cancer related to the MetS [8].

There is strong epidemiological evidence for an association between obesity and an increased risk of EAC [9] and a decreased risk of ESCC [10]. However, knowledge on the risk of esophageal cancer in relation to other MetS components is limited. Previous epidemiological studies have not demonstrated any clear evidence for an association between hyperglycemia and esophageal cancer overall, but a significant association in subanalysis of esophageal cancer with mortal outcome and esophageal cancer among men [11-13]. An association between blood lipids and esophageal cancer has been reported from one study that was not able to adjust for BMI or smoking habits [14]. It is noteworthy that all these studies share the methodological problem of using all esophageal cancer as endpoint. Considering the highly separate biological and epidemiological profile of EAC and ESCC [2], the lack of differentiation between EAC and ESCC significantly limits the scientific value of all these studies. Studies on the association between hypertension and EAC and ESCC are lacking.

The aim of the present study was to investigate the association between BMI, blood pressure, glucose, cholesterol, and triglycerides, both separately and combined, and the risk of EAC and ESCC in a large prospective cohort.

## Methods

### The metabolic syndrome and cancer project (Me-Can)

The Metabolic syndrome and Cancer project (Me-Can) was initiated in 2006 with the specific aim to investigate the association between components of the metabolic syndrome and overall- and site-specific cancer risk [15-22]. The Me-Can cohort consists of seven prospective cohorts in Austria, Norway and Sweden and has been described in detail previously [23]. In brief, after exclusion of subjects with unrealistic or missing baseline data or prevalent cancer diagnosis, the Me-Can cohort consists of data on 578 700 subjects (289 866 men and 288 834 women). Ethical clearance was obtained from each national ethical committee in Austria, Norway and Sweden.

### Assessment of exposure at baseline investigation

Study participants were subjected to health examination (s) between 1972 and 2005. The data collection procedure and details on measurement methods have been described in a previous publication [23]. In brief weight and height were measured without shoes with light indoor clothes in all cohorts. Blood pressure was measured in the supine or sitting position. Smoking habits were assessed by use of a self-administered questionnaire in all cohorts with the exception for the Austrian cohort (Vorarlberg Health Monitoring and Prevention Program), where the examining physician asked subjects about their smoking habits. Study subjects were not requested to fast before baseline examination in all cohorts, but fasting time before blood sampling was recorded in all subjects. Blood, plasma or serum levels of glucose, total cholesterol and triglycerides were analyzed.

### End-point assessment

The seven cohorts were linked to the respective National registers for cancer diagnosis, migration status (if available) and vital status. End of follow-up was 2006 in the Swedish cohorts, 2005 in the Norwegian cohorts and 2003 in the Austrian cohorts. Migration status was available in all cohorts except for the Australian cohort [23]. Subjects with an incident diagnosis of esophageal cancer were identified using the International Classification of Diseases (ICD), seventh edition (ICD-7), code 150. Morphology coding was available according to several different classification systems (C24 [24], Manual of Tumor Nomenclature (MOTNAC), ICD-Oncology (ICD-O) 1, ICD-O-2 and SNOMED) depending on study cohort and time of diagnosis. Only cases that were histologically verified were considered for the study.

### Statistical analysis

Quintile cut-off values were calculated separately in groups defined by cohort and sex for BMI and mid BP ((systolic BP + diastolic BP)/2) and in groups defined by cohort, sex and categories of fasting time (<4 hours, 4–8 hours and >8 hours) for glucose, cholesterol and triglycerides. In order to reduce the risk of a reverse causation, follow-up started one year after baseline examination. Subjects were followed until the date of diagnosis of esophageal cancer, death, migration, or end of follow-up, whichever occurred first. Incident cancers at other sites were not considered a criterion for censoring. Cox proportional hazards analysis was used to calculate relative risks (RR) with 95% confidence interval (CI) for EAC and ESCC related to quintile levels of all five components of the MetS. The proportional hazards assumption was met in all analyses as verified by log-log plots. Attained age was used as the underlying time scale. All models were stratified by cohort and by categories of birth-year (before 1923, 1923–1930, 1931–1938, 1939–1946, 1947–1954, 1955 and later). Relative risks were adjusted for age at baseline as a continuous variable and for sex, smoking status and quintile levels of BMI as categorical variables. We decided to include BMI in the final model due to the association between BMI and EAC and ESCC and the well-established association between BMI and other metabolic factors. The p*-*value for trend over quintiles refers to the Wald test of a linear risk estimate.

In order to make the variables comparable on a continuous scale and to create a combined MetS variable, the z-score standardization was used ((exposure level – mean)/standard deviation (SD)), resulting in a z-score of the exposures with a mean of 0 and a SD of 1. Glucose and triglycerides were log-transformed before standardization, as they were skewed and had outliers. BMI and mid blood pressure were standardized separately in groups defined by cohort and sex. Log (glucose), cholesterol and log (triglycerides) were standardized in groups based on cohort, sex and fasting time. The MetS score was calculated by summarizing the five individual z-scores before standardization. Cox proportional hazards regression was used to calculate RRs for EAC and ESCC related to the continuous z-score of the exposures. Again, attained age was used as time scale and the model was stratified by cohort and birth-year categories. In the analysis of the MetS, all estimates were subsequently adjusted for sex, age at baseline and smoking status. Relative risks related to the composite MetS score were adjusted for sex, age at baseline and smoking status. Additionally, the adjusted model of individual metabolic factors (BMI, mid blood pressure, glucose, cholesterol and triglycerides) included all metabolic factors at the same time.

RRs for EAC and ESCC were also assessed for all separate exposures as continuous variables (per five unit increment for BMI, per one unit increment of glucose, cholesterol and triglycerides and per 10 unit increment for mid BP). In this analysis, subjects with glucose levels > 10 mmol/l and triglycerides > 6 mmol/l were classified as outliers and excluded. RRs were adjusted for age at baseline, smoking status and all metabolic factors. Interactions between smoking and additional factors were tested by including cross-product terms in the regression models. A p-value of < 0.05 was considered to be indicative of a statistically significant interaction.

In addition to quintile categorization, BMI and blood pressure variables were further categorized according to World Health Organization (WHO) criteria for obesity [25] and European Society of Hypertension (ESH) and European Society of Cardiology (ESC) criteria for hypertension [26]. Underweight was defined as BMI ≤ 18.4, normal weight as BMI = 18.5-25.0, over weight as BMI = 25.0-29.9 and obesity as BMI ≥ 30 [25]. Blood pressure was classified as normal if systolic BP was < 140 and diastolic BP was < 90. Definition of severity of hypertension was grade I = systolic BP 140–159 or diastolic BP 90–99, grade II systolic BP = 160–179 or diastolic BP = 100–109 and grade III systolic BP ≥ 180 or diastolic BP ≥ 110. RR for EAC and ESCC related to WHO categories of BMI and ESH categories of hypertension were calculated using Cox’s proportional hazards regression stratifying and adjusting for the same variables as above.

#### Correction of a random error

In the analysis of exposure categorized in quintiles, regression dilution ratios (RDR) were calculated based on repeated health examinations in 133,820 subjects in the full Me-Can database in order to adjust RRs for random errors in the measurement of exposure variables at baseline [27,28], this process has been described in detail previously [13]. In brief, only measurements in the same cohort with the same fasting time before any incident cancer diagnosis were used. Correction of the RRs for RDRs was obtained by dividing the estimated parameter with RDR [exp (log (RR)/RDR)]. The estimated RDR were as follows; BMI 0.902, mid BP 0.544, glucose 0.278, cholesterol 0.657, triglycerides 0.505 and MetS 0.688.

When more than one variable with a random error was included in the analysis such as when z-score variables were analyzed, the RDR correction was not considered appropriate. In those situations a regression calibration model (RC) was used instead [27,29]. With this method, the exposure measured with error (the observed measurement) was replaced with a predicted value calculated from a regression model, again with age at baseline, birth year, fasting time, smoking status and time from baseline as fixed effects and cohort as random effect. The corrected measurement was then used in risk model estimation.

All statistical analyses were performed in SPSS Statistics 19.0 (Chicago, Illinois) except calculation of RDR and regression calibration that was calculated in R, version 2.7.2.

## Results

Baseline characteristics for the Me-Can cohort and cases of EAC and ESCC are presented in Table 1. Fifty percent of the participants were male and 50% were female, mean age at baseline was 44.0 years, mean BMI was 25.3, 27.7% were current smokers, 27.4% were former smokers and 44.6% were never-smokers. Mean time of follow-up was 12 years.

**Table 1:** Baseline characteristics

		**Cases**				**Total cohort**^**1**^
		**Adenocarcinoma**	**Squamous cell carcinoma**	**Other or undifferentiated morphology**	**All cases**	
Subjects, n		114	184	26	324	577259
Sex	Male	102 (89.5)	144 (78.3)	22 (84.6)	268 (82.7)	288930 (50.1)
	Female	12 (10.5)	40 (21.7)	4 (15.4)	56 (17.3)	288329 (49.9)
Age at baseline, mean (SD)		49.6 (10.1)	51.0 (10.9)	51.2 (10.1)	50.5 (10.5)	44.0 (11.7)
Cohort, n (%)	Oslo	13 (11.4)	37 ((20.1)	5 (19.2)	55 (17.0)	16714 (2.9)
	NCS	31 (27.2)	29 (15.8)	4 (15.4)	64 (19.8)	50922 (8.8)
	CONOR	10 (8.8)	14 (7.6)	3 (11.5)	27 (8.3)	109403 (19.0)
	40-y	5 (4.4)	6 (3.3)	1 (3.8)	12 (3.7)	128742 (22.3)
	VHM&PP	16 (14.0)	40 (21.7)	5 (19.2)	61 (18.8)	159444 (27.6)
	VIP	16 (14.0)	8 (4.3)	1 (3.8)	25 (7.7)	79360 (13.7)
	MPP	23 (20.2)	50 (27.2)	7 (26.9)	80 (24.7)	32674 (5.7)
Fasting time, n (%)	< 4 hrs	43 (37.7)	64 (34.8)	8 (30.8)	115 (35.5)	242246 (42.0)
	4-8 hrs	14 (12.3)	14 (7.6)	5 (19.2)	33 (10.2)	57409 (9.9)
	> 8 hrs	57 (50.0)	106 (57.6)	13 (50.0)	176 (54.3)	277604 (48.1)
BMI, mean (SD)		27.1 (3.8)	24.0 (3.3)	26.0 (4.2)	25.3 (3.8)	25.3 (4.0)
Mid BP^2^ mmHg, mean (SD)		110.6 (12.9)	112.4 (15.3)	111.9 (12.7)	111.7 (14.3)	104.4 (13.7)
Glucose mmol/l, median (IQR)		5.4 (4.8-5.4)	5.3 (4.7-5.3)	5.1 (4.6-5.6)	5.3 (4.7-5.9)	5.1 (4.6-5.6)
Cholesterol mmol/l, mean (SD)		6.1 (1.1)	6.2 (1.1)	6.4 (1.3)	6.2 (1.1)	5.7 (1.2)
Triglycerides mmol/l, median (IQR)		1.63 (1.11-2.43)	1.44 (1.02-2.11)	1.82 (1.12-2.80)	1.57 (1.05-2.33)	1.29 (0.91-1.91)
Smoking status, n (%)	Never	25 (21.9)	29 (15.8)	4 (15.4)	58 (17.9)	257721 (44.6)
	Former	36 (31.6)	25 (13.6)	5 (19.2)	66 (20.4)	158358 (27.4)
	Current	52 (45.6)	129 (70.1)	17 (65.4)	198 (61.1)	159624 (27.7)
	Missing	1 (0.9)	1 (0.5)	0	2 (0.6)	1556 (0.3)

^1^After exclusion of 1441 subjects with a follow-up less than 1 year.

^2^Mid blood pressure = [(systolic blood pressure + diastolic blood pressure)/2] mm Hg).

*Abbreviations: Oslo* The Oslo study I cohort, *NCS* The Norwegian County Study, *CONOR* The cohort of Norway, *40-y:* The Age 40-programme, *VHM&PP* The Vorarlberg Health Monitoring and Prevention Program, *VIP* The Västerbotten Intervention Project, *MPP* The Malmö Preventive Project, *BMI* body mass index (kg/m^2^).

### Body mass index and risk of esophsageal adenocarcinoma

There was a statistically significant association between BMI and the risk of EAC with a clear dose–response relationship over quintiles (adjusted RR for top versus bottom quintile of BMI was 7.34 (95% CI 2.88-18.68) and corresponding RDR corrected adjusted RR was 9.18 (95% CI, 3.24-25.96)) (Table 2). This association was also statistically significant when BMI was standardized into z-scores (RR 1.64 (95% CI, 1.30-2.07) per one unit increase of calibrated z-score) (Table 3). The RRs of EAC related to WHO categories of BMI were 3.29 (95% CI, 1.82-5.95) for BMI ≥30 and 2.32 (95% CI, 1.51-3.57) for BMI 25.0-29.9, adjusted for sex, age and smoking status using subjects with BMI of 18.5-24.9 as reference category (Table 4). There was no interaction between smoking status and investigated metabolic factors as risk factors for EAC with the exception for an interaction between triglycerides and former (versus never) smokers (p = 0.01) (Table 5). BMI was significantly associated with the risk of EAC among current and former smokers and there was a non-significant tendency towards an association among never smokers (Table 5).

**Table 2:** Relative risks for esophageal cancer related to different metabolic risk factors in quintiles

**Exposure**			**Adenocarcinoma**				**Squamous cell carcinoma**			
	**Quintile**	**Mean (SD)**	**Cases (n)**	**Age and cohort stratified RR**	**Adjusted RR**^**1**^	**Adjusted, RDR corrected RR**^**1**^	**Cases (n)**	**Age and cohort stratified RR**	**Adjusted RR**^**1**^	**Adjusted, RDR corrected RR**^**1**^
BMI	1	20.7 (1.5)	5	1.00	1.00	1.00	55	1.00	1.00	1.00
	2	23.0 (1.1)	18	3.13 (1.16–8.44)	3.37 (1.25–9.10)	3.86 (1.28–11.66)	29	0.44 (0.28–0.70)	0.50 (0.32–0.79)	0.47 (0.28–0.77)
	3	24.7 (1.0)	18	2.81 (1.04–7.60)	3.17 (1.17–8.57)	3.61 (1.19–10.91)	46	0.62 (0.42–0.92)	0.76 (0.51–1.12)	0.73 (0.47–1.14)
	4	26.8 (1.0)	31	4.41 (1.71–11.39)	5.19 (2.00–13.42)	6.24 (2.17–17.97)	30	0.37 (0.23–0.57)	0.46 (0.30–0.72)	0.42 (0.26–0.70)
	5	31.3 (3.3)	42	5.96 (2.34–15.16)	7.34 (2.88–18.68)	9.18 (3.24–25.96)	24	0.29 (0.18–0.47)	0.38 (0.23–0.62)	0.34 (0.20–0.58)
*P*trend				<0.0001	<0.0001	<0.0001		<0.0001	<0.0001	
*Adjusted RR per increment of 5*^*1*^				1.64 (1.35–2.00)	1.78 (1.45–2.17)			0.56 (0.44–0.70)	0.62 (0.50–0.79)	
Mid blood pressure, mmHg	1	88 (5.7)	14	1.00	1.00	1.00	21	1.00	1.00	1.00
	2	97 (4.1)	18	1.12 (0.56–2.25)	0.99 (0.49–2.00)	0.99 (0.27–3.58)	30	1.29 (0.74–2.26)	1.47 (0.84–2.58)	2.04 (0.73–5.70)
	3	103 (3.8)	22	1.24 (0.63–2.44)	1.15 (0.58–2.27)	1.29 (0.37–4.50)	23	0.87 (0.48–1.58)	1.14 (0.63–2.08)	1.28 (0.43–3.83)
	4	110 (4.1)	25	1.13 (0.58–2.19)	1.01 (0.52–1.98)	1.02 (0.30–3.50)	49	1.55 (0.93–2.61)	2.27 (1.35–3.83)	4.51 (1.72–11.81)
	5	125 (10.4)	35	1.32 (0.70–2.51)	1.09 (0.57–2.10)	1.17 (0.35–3.90)	60	1.59 (0.95–2.66)	2.60 (1.54–4.39)	5.79 (2.21–15.20)
*P*trend				0.44	0.81			0.035	<0.0001	
*Adjusted RR per increment of 10 mmHg*^*1*^				1.09 (0.95–1.25)	1.03 (0.89–1.19)			1.17 (1.05–1.29)	1.30 (1.17–1.44)	
Glucose, mmol/L	1	4.1 (0.5)	19	1.00	1.00	1.00	34	1.00	1.00	1.00
	2	4.7 (0.3)	21	1.06 (0.57–1.97)	1.07 (0.57–2.01)	1.29 (0.14–12.21)	33	0.96 (0.59–1.55)	1.04 (0.64–1.69)	1.16 (0.20–6.55)
	3	5.1 (0.3)	20	0.96 (0.51–1.81)	0.95 (0.51–1.80)	0.85 (0.09–8.32)	38	1.07 (0.69–1.70)	1.20 (0.75–1.91)	1.90 (0.35–10.27)
	4	5.5 (0.3)	28	1.28 (0.71–2.31)	1.32 (0.73–2.38)	2.69 (0.32–22.70)	31	0.88 (0.54–1.44)	1.02 (0.62–1.68)	1.08 (0.18–6.43)
	5	6.8 (1.9)	26	1.07 (0.59–1.95)	1.04 (0.57.1.90)	1.14 (0.13–10.12)	48	1.21 (0.77–1.89)	1.44 (0.92–2.27)	3.76 (0.74–19.17)
*P*trend				0.62	0.70			0.50	0.14	
*Adjusted RR per increment of 1 mmol/l*^*2,3*^				1.14 (0.93–1.38)	1.12 (0.92–1.38)			1.16 (0.99–1.35)	1.22 (1.04–1.42)	
Cholesterol, mmol/L	1	4.2 (0.5)	13	1.00	1.00	1.00	22	1.00	1.00	1.00
	2	5.0 (0.3)	25	1.60 (0.82–3.15)	1.48 (0.75–2.91)	1.82 (0.65–5.08)	30	1.09 (0.63–1.89)	1.08 (0.62–1.88)	1.13 (0.49–2.62)
	3	5.6 (0.3)	18	1.01 (0.49–2.08)	0.93 (0.45–1.92)	0.90 (0.30–2.69)	36	1.16 (0.68–1.99)	1.20 (0.70–2.05)	1.32 (0.59–2.99)
	4	6.2 (0.3)	27	1.34 (0.68–2.63)	1.22 (0.62–2.40)	1.36 (0.49–3.80)	44	1.26 (0.75–2.12)	1.32 (0.78–2.21)	1.52 (0.69–3.35)
	5	7.4 (0.8)	31	1.34 (0.69–2.62)	1.22 (0.63–2.37)	1.35 (0.49–3.72)	51	1.32 (0.79–2.21)	1.42 (0.85–2.37)	1.70 (0.78–3.71)
*P*trend				0.68	0.86			0.22	0.12	
*Adjusted RR per increment of 1 mmol/l*^*2*^				0.99 (0.84–1.17)	0.94 (0.80–1.10)			1.06 (0.94–1.21)	1.08 (0.95–1.22)	
Triglycerides, mmol/L	1	0.72 (0.17)	14	1.00	1.00	1.00	27	1.00	1.00	1.00
	2	1.03 (0.21)	18	1.20 (0.60–2.42)	1.03 (0.51–2.07)	1.05 (0.26–4.22)	39	1.28 (0.78–2.09)	1.26 (0.77–2.06)	1.57 (0.59–4.17)
	3	1.33 (0.29)	16	0.98 (0.48–2.02)	0.78 (0.38–1.60)	0.61 (0.15.2.55)	34	1.05 (0.63–1.74)	1.05 (0.63–1.75)	1.11 (0.40–3.04)
	4	1.77 (0.42)	33	1.95 (1.04–3.65)	1.42 (0.75–2.69)	2.00 (0.56–7.09)	39	1.14 (0.70–1.87)	1.22 (0.74–2.01)	1.48 (0.55–3.99)
	5	3.12 (1.54)	28	1.60 (0.84–3.06)	1.05 (0.54–2.05)	1.11 (0.30–4.15)	44	1.24 (0.77–2.02)	1.45 (0.87–2.39)	2.07 (0.77–5.61)
*P*trend				0.038	0.51			0.58	0.22	
*Adjusted RR per increment of 1 mmol/l*^*2,4*^				1.35 (1.12–1.61)	1.13 (0.93–1.38)			1.15 (0.98–1.34)	1.19 (1.01–1.40)	

All analyses were stratified by study cohort and birth-year category. See text for correction of regression dilution bias.

^1^Adjusted for sex, age at baseline (continuous) smoking status and quintiles of BMI. ^2^Adjusted for age at baseline (continuous), smoking status, quintiles of BMI and fasting time. ^3^Outliers >10 mmol/l are excluded. ^4^Outliers >6 mmol/l are excluded.

*Abbreviations: CI* confidence interval, *RR* relative risk, *SD* standard deviation, *BMI* body mass index, *Mid BP:* mid blood pressure, *RDR* regression dilution ratio.

**Table 3:** Risk for esophageal cancer for continuous z-scores of single metabolic factors and the metabolic syndrome

***Exposure***	***Adenocarcinoma (n = 114)***			***Squamous cell carcinoma (n = 184)***		
	***RR model 1***^***1***^	***RR model 2***^***2***^	***Regression calibrated RR***^***3***^	***RR model 1***^***1***^	***RR model 2***^***2***^	***Regression calibrated RR***^***3***^
BMI	1.57 (1.35–1.83)	1.58 (1.34–1.87)	1.64 (1.30–2.07)	0.69 (0.58–0.81)	0.60 (0.50–0.72)	0.50 (0.40–0.63)
Mid blood pressure	1.10 (0.92–1.32)	0.99 (0.81–1.21)	0.95 (0.66–1.39)	1.28 (1.13–1.46)	1.40 (1.23–1.60)	1.77 (1.37–2.29)
Glucose	1.04 (0.86–1.24)	0.97 (0.80–1.17)	0.99 (0.49–1.99)	1.11 (0.98–1.27)	1.11 (0.97–1.27)	1.40 (0.85–2.31)
Cholesterol	1.02 (0.84–1.23)	0.95 (0.77–1.17)	0.92 (0.66–1.29)	1.07 (0.92–1.24)	1.08 (0.92–1.26)	1.10 (0.85–1.41)
Triglycerides	1.20 (0.99–1.45)	1.05 (0.85–1.30)	1.13 (0.71–1.80)	0.99 (0.86–1.16)	1.05 (0.88–1.24)	1.03 (0.71–1.49)
MetS^4^	1.36 (1.13–1.64)	–	1.56 (1.19–2.05)	1.06 (0.91–1.24)	–	1.09 (0.87–1.36)

Relative risk with 95% confidence interval for esophageal adenocarcinoma and squamous cell carcinoma for continuous z-score of single metabolic factors and the combined z-score for the metabolic syndrome.

^1^Relative risk from Cox regression models, with attained age as time scale, stratified by cohort and categories of birth year. Adjusted for sex, age at baseline and smoking.

^2^As model 1 but in addition adjusted for the z-score of analyzed factors i.e. BMI, mid BP, glucose, cholesterol and triglycerides.

^3^As model 2 and corrected by regression calibration, see text.

^4^Z score for MetS is adjusted for sex, age at baseline and smoking status. Corrected for regression dilution bias, see text.

*Abbreviations: MetS* metabolic syndrome, *BMI* body mass index, *RR* relative risk.

**Table 4:** Risk for esophageal cancer in relation to clinical categories of obesity and hypertension

**Exposure**	**Category**	**Adenocarcinoma**		**Squamous cell carcinoma**	
		**Crude RR**	**Adjusted RR**^**1**^	**Crude RR**	**Adjusted RR**^**2**^
BMI in WHO categories	≤ 18.4	-	-	1.68 (0.62–4.56)	1.54 (0.56–4.19)
	18.5–25.0	Reference	Reference	Reference	Reference
	25.0–29.9	2.53 (1.66–3.88)	2.32 (1.51–3.57)	0.65 (0.47–0.90)	0.67 (0.49–0.93)
	≥ 30	2.69 (1.50–4.85)	3.29 (1.82–5.95)	0.39 (0.19–0.77)	0.47 (0.24–0.94)
	≥ 25	2.57 (1.70–3.88)	2.47 (1.63–3.74)	0.60 (0.44–0.82)	0.64 (0.47–0.87)
ESH/ESC hypertension criteria^3^	Normal	Reference	Reference	Reference	Reference
	Grade 1	1.13 (0.74–1.72)	0.90 (0.59–1.32)	1.34 (0.96–1.87)	1.61 (1.15–2.26)
	Grade 2	1.47 (0.84–2.57)	1.11 (0.63–1.96)	1.42 (0.89–2.26)	1.98 (1.23–3.17)
	Grade 3	0.93 (0.33–2.62)	0.66 (0.23–1.87)	1.96 (1.10–3.59)	2.95 (1.62–5.37)
	Grade 1–3	1.19 (0.81–1.74)	0.93 (0.63–1.37)	1.41 (1.04–1.91)	1.77 (1.30–2.42)
	*P*trend	0.39	0.72	0.01	<0.0001

Relative risks with 95% confidence interval for incident esophageal adenocarcinoma and squamous cell carcinoma related to BMI and blood pressure categorized in clinical criteria for obesity and hypertension. All analyses were stratified by study cohort and birth-year category.

^1^Adjusted for sex, age at baseline (continuous) and smoking status.

^2^Adjusted for sex, age at baseline (continuous), smoking status and BMI (continuous).

^3^European Society of Hypertension (ESH) and European Society of Cardiology (ESC) criteria for hypertension: Normal blood pressure (BP): systolic BP < 140 and diastolic BP < 90. Grade I hypertension: systolic BP 140–159 or diastolic BP 90–99, grade II: systolic BP = 160–179 or diastolic BP = 100–109 and grade III: systolic BP ≥ 180 or diastolic BP ≥ 110.

*Abbreviations: RR* relative risk, *CI* confidence interval, *BMI* body mass index, *WHO* World Health Organization, *ESH* European Society of Hypertension, *ESC* European Society of Cardiology.

**Table 5:** Risk for esophageal cancer in relation to metabolic risk factors stratified for smoking

**Smoking status**	**Exposure**	**Adenocarcinoma**				**Squamous cell carcinoma**			
		**RR model 1**^**1**^	**RR model 2**^**2**^	**Regression calibrated RR**^**3**^	**Interaction**^**4**^**p-value**	**RR model 1**^**1**^	**RR model 2**^**2**^	**Regression calibrated RR**^**3**^	**Interaction**^**4**^**p-value**
Never smoker	BMI	1.22 (0.83–1.77)	1.30 (0.87–1.94)	1.28 (0.75–2.19)		0.72 (0.47–1.09)	0.67 (0.43–1.06)	0.61 (0.35–1.07)	
	Mid BP	1.08 (0.73–1.60)	1.11 (0.73–1.67)	1.15 (0.52–2.53)		1.23 (0.89–1.70)	1.35 (0.97–1.89)	1.75 (0.93–3.30)	
	Glucose	1.19 (0.86–1.65)	1.22 (0.86–1.71)	2.00 (0.55–7.36)		1.03 (0.75–1.43)	1.01 (0.71–1.43)	0.91 (0.25–3.32)	
	Cholesterol	0.81 (0.53–1.26)	0.82 (0.50–1.34)	0.82 (0.38–1.18)		0.93 (0.63–1.37)	0.90 (0.59–1.37)	0.84 (0.43–1.65)	
	Triglycerides	0.77 (0.49–1.20)	0.71 (0.44–1.17)	0.45 (0.16–1.27)		0.97 (0.66–1.44)	1.07 (0.69–1.65)	1.10 (0.43–2.83)	
	Mets	1.07 (0.69–1.65)	-	1.10 (0.58–2.08)		0.98 (0.66–1.47)	-	0.98 (0.54–1.75)	
Former smoker	BMI	1.87 (1.49–2.35)	1.89 (1.44–2.47)	2.14 (1.44–3.18)	0.07	0.91 (0.59–1.40)	0.78 (0.49–1.24)	0.62 (0.35–1.10)	0.59
	Mid BP	1.14 (0.83–1.56)	0.96 (0.68–1.36)	0.91 (0.47–1.77)	0.28	1.52 (1.09–2.11)	1.53 (1.09–2.16)	1.93 (0.99–3.75)	0.47
	Glucose	0.86 (0.61–1.23)	0.73 (0.51–1.06)	0.43 (0.11–1.64)	0.46	1.30 (0.98–1.72)	1.27 (0.94–1.71)	2.80 (0.99–7.93)	0.14
	Cholesterol	1.15 (0.83–1.58)	1.04 (0.72–1.50)	1.01 (0.56–1.80)	0.14	1.14 (0.77–1.69)	1.12 (0.73–1.70)	1.21 (0.61–2.41)	0.56
	Triglycerides	1.54 (1.11–2.13)	1.28 (0.88–1.85)	1.96 (0.87–4.39)	0.01	1.02 (0.68–1.52)	0.92 (0.59–1.45)	0.73 (0.28–1.94)	0.68
	MetS	1.60 (1.16–2.21)	-	1.99 (1.25–3.16)	0.08	1.39 (0.94–2.04)	-	1.61 (0.92–2.82)	0.21
Current smoker	BMI	1.54 (1.22–1.94)	1.52 (1.18–1.95)	1.53 (1.09–2.16)	0.35	0.63 (0.52–0.77)	0.55 (0.45–0.68)	0.46 (0.35–0.61)	0.41
	Mid BP	1.13 (0.87–1.48)	1.02 (0.76–1.37)	1.03 (0.59–1.80)	0.71	1.26 (1.07–1.48)	1.39 (1.18–1.63)	1.75 (1.28–2.38)	0.76
	Glucose	1.03 (0.77–1.36)	0.95 (0.71–1.29)	0.96 (0.32–2.88)	0.97	1.08 (0.92–1.27)	1.09 (0.92–1.29)	1.26 (0.67–2.37)	0.83
	Cholesterol	1.03 (0.78–1.35)	0.93 (0.69–1.27)	0.89 (0.54–1.47)	0.36	1.09 (0.92–1.29)	1.11 (0.92–1.33)	1.14 (0.84–1.53)	0.44
	Triglycerides	1.22 (0.93–1.61)	1.09 (0.80–1.49)	1.24 (0.63–2.43)	0.07	1.00 (0.83–1.19)	1.07 (0.87–1.31)	1.09 (0.70–1.69)	0.90
	MetS	1.37 (1.04–1.81)	-	1.58 (1.05–2.37)	0.38	1.01 (0.84–1.22)	-	1.02 (0.78–1.34)	0.96

Relative risk for esophageal cancer in relation to continuous z-score of metabolic factors and the composite metabolic syndrome score, stratified for smoking status.

^1^Relative risk from Cox regression models with attained age as time scale, stratified by cohort and categories of birth year within the model, adjusted for sex and age at baseline.

^2^As model 1 but in addition adjusted for all metabolic factors.

^3^Adjusted as model 2. Corrected by regression calibration, see text.

^4^Each metabolic factor multiplied by smoking status (current or former) was entered in the analysis as an interaction term. Adjusted as model.

*Abbreviations: RR* relative risk, *MetS* metabolic syndrome, *BMI* body mass index, *Mid BP* mid blood pressure.

### Other metabolic risk factors and the risk of esophsageal adenocarcinoma

Mid BP, glucose, cholesterol and triglycerides were not associated with the risk of EAC (Table 2 and 3). There was a statistically significant association between the composite MetS score and the risk of EAC (RR 1.56 (95% CI, 1.19-2.05) per one unit increase of the composite MetS score) (Table 3).

### Body mass index and risk of esophageal squamous cell carcinoma

Higher BMI was statistically significantly associated with a decreased risk of ESCC (adjusted RR for top versus bottom quintile of BMI was 0.38 (95% CI, 0,23-0,62) and corresponding RDR corrected adjusted RR was 0.34 (95% CI, 0.20-0.58) (Table 2). When BMI classified into WHO categories was analyzed, a negative dose–response relationship was observed. The adjusted RR was 0.67 (95% CI, 0.49-0.93) for BMI 25–29.9, and 0.47 (95% CI, 0.24-0.94) for BMI ≥30, using BMI 18.5-24.9 as reference category (Table 4). This association was statistically significant also when BMI was analyzed as a continuous variable (RR 0.62 (95% CI 0.50-0.79) per increment of 5 in BMI) (Table 2), and standardized into z-scores (RR 0.50 (95% CI, 0.40-0.63) per one unit increase in z-score) (Table 3). There was no interaction between smoking status and any of the investigated metabolic factors as risk factors for ESCC. The association between high BMI and decreased risk of ESCC was statistically significant in current smokers. In never and former smokers there was a similar trend that, however, did not reach statistical significance (Table 5).

### Blood pressure and the risk of esophageal squamous cell carcinoma

Higher mid BP was associated with an increased risk of ESCC. The adjusted RR for ESCC was 2.60 (95% CI 1.54-4.39) for top versus bottom quintile of mid BP and corresponding RDR corrected adjusted RR was 5.79 (95% CI, 2.21-15.20) (Table 2). In the analysis of mid BP as a continuous variable the RR for ESCC was 1.30 (95% CI, 1.17-1.44) (Table 2) per increment of 10 mmHg and RR for mid BP standardized into z-score was 1.77 (95% CI, 1.37-2.29) per one unit of z-score increment (Table 3). Using ESH/ESC criteria for hypertension revealed a clear dose–response relationship between hypertension grade and risk of ESCC with a RR of 1.61 (95% CI, 1.15-2.26), 1.98 (95% CI 1.23-3.17) and 2.95 (95% CI, 1.62-5.37) for grade I, II and III hypertension respectively (p-value for trend <0.001) (Table 4). There was no statistically significant interaction between smoking and mid BP (Table 5). Estimates of RR for ESCC related to mid BP z-scores were similar in all strata of smoking status, albeit statistically significant only in current smokers (Table 5).

### Other metabolic risk factors and the risk of esophageal squamous cell carcinoma

There was no association between glucose, cholesterol or triglycerides and risk of ESCC (Table 2 and 3), with the exception for a borderline significant association between triglycerides as a continuous variable in the fully adjusted model (RR 1.19 (95% CI, 1.01-1.40) per increment of 1 mmol/l). The composite variable for the MetS was not statistically significantly associated with risk of ESCC (RR 0.92 (95% CI, 0.79-1.08) per 1 unit increment of the composite MetS score) (Table 3).

## Discussion

The association between metabolic factors and the risk of the two dominating types of esophageal cancer, EAC and ESCC, was investigated in this large prospective cohort study. There was a strong association between high BMI and an increased risk of EAC and a decreased risk of ESCC. Mid BP was associated with an increased risk of ESCC. The composite MetS score was associated with an increased risk of EAC but not with the risk of ESCC.

The association between overweight and EAC is known from several previous studies [9,30-33]. In a meta analysis from 2006, Kubo et al. reported a pooled OR of 1.7 (95% CI, 1.6-1.9) for EAC related to overweight and obesity compared to normal weight [9]. Smith et al. reported a pooled RR of 1.54 (95% CI, 1.39-1.71) per increment of 5 in BMI for all identified case–control studies and Engeland et al. reported a RR of 1.53 (95% CI, 1.30-1.79) in a Norwegian cohort that is partly overlapping the Norwegian Me-Can cohort. The association between BMI and the risk of EAC in the present study is slightly stronger than those reported in previous meta analyses with a RR of 1.78 (95% CI 1.45-2.17) per increment of 5 in BMI and a RR of 2.47 (95% CI, 1.63-3.74) for overweight and obesity compared to normal weight. Misclassification of EAC as ESCC can be expected to attenuate risk estimates given the known inverse association between BMI and ESCC. It is possible that this kind of bias has been less important in the present study, considering the high quality of the national cancer registries that were used.

Two different causal links between obesity and EAC can be hypothesized. One possible mechanism is through and increased risk for gastroesophageal reflux. Obesity is associated with an increased risk of gastro-esophageal reflux [34] which in turn is associated with the development of intestinal metaplasia in the distal esophagus, i.,e Barrett’s esophagus [35], a pre malignant condition associated with the risk of EAC [36].

Another possible mechanism for the association between obesity and EAC is through a hormonal and/or metabolic systemic disequilibrium related to the MetS [37]. The MetS has been demonstrated to be associated with several site-specific cancers, including liver, colorectal, breast, pancreatic, urinary bladder, and endometrial cancer [8]. The mechanisms for the association between the MetS and cancer are not fully characterized. Chronic low-grade inflammation, high levels of trophic hormones (ie insulin and insulin-like growth factor) or lifestyle-related factors related to the MetS have been proposed as putative mechanisms [8].

In the present study, we found a statistically significant association between the surrogate score for the MetS and the risk of EAC. However, BMI was the only metabolic factor with a statistically significant association with the risk of EAC. Therefore, we consider that our findings suggest that obesity leading to gastro-esophageal reflux and esophageal dysplasia may be the more important mechanism. Nevertheless, this does not exclude a role for metabolic state related to the MetS for the development of EAC. High leptin levels and low levels of high molecular weight adipnectin have been associated with an increased risk for progression from Barrett’s esophagus to EAC after adjustment for relevant other risk factors, including BMI [38].

The inverse association between BMI and the risk for ESCC demonstrated in the present study has been observed in several previous studies [10,39,40]. In a meta-analysis by Smith et al., data from 3 cohort studies were pooled and the RR for ESCC per increment of 5 in BMI was estimated to 0.69 (95% CI, 0.63-0.75) [10]. The inverse association between BMI and risk of ESCC in the present study was similar to the above-mentioned studies with a RR of 0.62 (95% CI, 0.50-0.79) per increment of 5 in BMI. Despite an inverse association between BMI and ESCC, Steffen et al. recently observed a positive association between waist-hip-ratio and risk of ESCC in a model adjusted for BMI [33]. We had no possibility to investigate the association between waist-hip ratio and ESCC risk since this information was not available in the Me-Can cohort. The association between BMI and ESCC was only statistically significant in current smokers and data on smoking dose was not available for the adjusted analysis. As a consequence, even though the analysis was adjusted for smoking status, smoking dose may have been a confounder in the association between BMI and the risk of ESCC found in this study, considering that smoking is associated with low BMI and a well-established risk factor for ESCC [41].

We found a strong and dose dependent association between mid BP and risk of ESCC. However, alcohol consumption is a known risk factor for hypertension [42] and has also consistently been associated with the risk for ESCC [5]. It is therefore possible that alcohol consumption is a confounder in the observed association between hypertension and ESCC. An increased risk of esophageal cancer in general related to hypertension diagnosed below the age of 60 years was recently reported in a study from the Saskatchewan Health database [43], but we know of no studies to date, exploring the possible association between hypertension and ESCC or EAC. The association between hypertension and cancer in general has been explored in previous studies finding either no [44] or a modest positive association [45,46].

An association between high blood glucose and an increased risk of cancer overall has been reported in several prospective studies [11,13]. Proposed mechanisms for this association include a direct mitotic effect of insulin-like growth factor and oxidative stress related to hyperglycemia [47]. We did not find any association between serum glucose and EAC and no significant association between glucose and ESCC except for when glucose was entered as a continuous variable. Previous studies on the association between esophageal cancer and serum glucose have been conflicting, demonstrating no association for overall esophageal cancer in most studies [11-13] but positive associations in subgroups of hyperglycemic subjects (i.e. men with diabetes [12,48], fatal esophageal cancer [13] or fatal esophageal cancer among men [11]). A limitation to all these studies is that there was no differentiation between EAC and ESCC. The association between diabetes and esophageal cancer has recently been investigated in a metaanalysis where an increased risk was found among men but not women [49]. Subanalysis of three studies separating EAC from ESCC revealed that diabetes was associated with EAC [49].

Well-designed studies on the association between blood lipids and different subtypes of esophageal cancer are lacking. A positive association between esophageal cancer and both triglycerides and low-density lipoprotein cholesterol/high-density lipoprotein cholesterol has been reported in a recent cohort study [14]. However, BMI and smoking was not adjusted for in that study. We observed a statistically significant trend over triglyceride quintiles and risk of EAC in crude analysis that disappeared when BMI was adjusted for, indicating that BMI may have been a confounder in the above-mentioned study [14]. We did not find any association between triglycerides or cholesterol and the risk of ESCC. To the best of our knowledge, there is no evidence for such an association in the literature.

Major strengths of the present study are the prospective design and the large size of the cohort. The large proportion of subjects with repeated measurements in the cohort enabled us to adjust for random error in measurement of metabolic factors. National cancer registries in Sweden, Norway and Austria have a previously been demonstrated to be highly accurate [19,50,51] assuring a high quality in the end-point assessment. The possibility to differentiate between EAC and ESCC was another important strength. As demonstrated in the present study, these two types of esophageal cancer have very different risk factor profile and the value of previous studies analyzing all esophageal cancer together can be questioned. Differentiation between distal EAC and adenocarcinoma of the gastric cardia may in some cases be difficult and some misclassification of gastric cardia cancers as EAC has most probably occurred in this study. However, adenocarcinoma of the gastric cardia and EAC are associated with BMI and smoking in a similar manner [39] and limited misclassification between these cancers will not have any major impact on investigated risk factors. Differences in measurement methods between the different cohorts is a limitation to the study that we have tried to overcome by using cohort specific cut-offs for quintiles and z-score standardization. Another shortcoming of the study is the lack of information on ethnicity, considering the previously demonstrated association between EAC and white race [3]. The above-mentioned use of cohort specific cut-offs and z-score standardization and the relative homogeneity of the individual cohorts have probably reduced the impact of this bias. Information on smoking habits was limited. Subjects could be classified as never, current or former smokers but quantitative data was lacking. In order to compensate for this, positive findings were reanalyzed in separate strata of smoking habits. The associations between BMI and EAC, BMI and ESCC and Mid BP and ESCC were homogenous, even though not statistically significant, in all strata of smoking habits including never smokers. The possibility of a type I error should also be kept in mind since multiple comparisons were made.

## Conclusions

This study confirms the previously described association between a high BMI and an increased risk of EAC. A significant association was found between the surrogate score for the MetS and the risk of EAC. However, considering that no other metabolic risk factors were associated with EAC risk, we hypothesize that it is not the hormonal metabolic state related to the MetS that is the probable cause of the association between BMI and EAC. More likely, other factors associated with obesity such as increased risk of gastro esophageal reflux disease may play a role. We were also able to confirm the previously described negative association between BMI and ESCC and we found a positive association between blood pressure and ESCC that, to the best of our knowledge, has never been described before. However, this finding has to be confirmed in studies where proper adjustment for alcohol consumption is possible.

## Abbreviations

EAC: Esophageal adenocarcinoma; ESCC: Esophageal squamous cell carcinoma; MetS: Metabolic syndrome; BMI: Body mass index; BP: Blood pressure; WHO: World Health Organization; ESH: European Society of Hypertension; ESC: European Society of Cardiology; RR: Relative risk; CI: Confidence interval; Me-Can: The metabolic syndrome and cancer risk project; RDR: Regression dilution ratio; RC: Regression calibration.

## Competing interests

The authors declare that they have no competing of interests.

## Authors’ contributions

BL and JM have full access to all of the data in the study and take responsibility for the integrity of the data and the accuracy of the data analysis. Study concept and design: BL, DJ, TS, HC, TB, MA, CH, AE, GH, GN, HJ, RS, HU, ST, PS, JM. Acquisition of data: BL, DJ, TS, HC, TB, MA, CH, AE, GH, GN, HJ, RS, HU, ST, PS, JM. Drafting of the manuscript: BL. Critical revision of the manuscript for important intellectual content: BL, DJ, TS, HC, TB, MA, CH, AE, GH, GN, HJ, RS, HU, ST, PS, JM. Statistical analysis: BL, HJ, JM. Obtaining funding: JM, PS. Study supervision: JM, PS. All authors read approved the final manuscript.

## Pre-publication history

The pre-publication history for this paper can be accessed here:

http://www.biomedcentral.com/1471-2407/14/103/prepub
